# The role of mesolevel characteristics of the health care system and socioeconomic factors on health care use – results of a scoping review

**DOI:** 10.1186/s12939-024-02122-6

**Published:** 2024-02-23

**Authors:** Philip Bammert, Wiebke Schüttig, Anna Novelli, Iryna Iashchenko, Jacob Spallek, Miriam Blume, Katharina Diehl, Irene Moor, Nico Dragano, Leonie Sundmacher

**Affiliations:** 1 Chair of Health Economics, Technical University of Munich, Munich, Germany; 2 Department of Public Health, Brandenburg University of Technology Cottbus-Senftenberg, Senftenberg, Germany; 3 Lausitz Center for Digital Public Health, Brandenburg University of Technology, Senftenberg, Germany; 4 Department of Epidemiology and Health Monitoring, Robert-Koch-Institute, Berlin, Germany; 5 Department of Medical Informatics, Biometry and Epidemiology, Friedrich-Alexander-Universität Erlangen-Nürnberg, Erlangen, Germany; 6 Institute of Medical Sociology, Interdisciplinary Center for Health Sciences, Medical Faculty, Martin Luther University Halle-Wittenberg, Halle, Germany; 7 Institute of Medical Sociology, Centre for Health and Society, University Hospital and Medical Faculty, University of Duesseldorf, Duesseldorf, Germany

**Keywords:** Healthcare use, Inequities, Access, Mesolevel, Scoping review

## Abstract

**Background:**

Besides macrolevel characteristics of a health care system, mesolevel access characteristics can exert influence on socioeconomic inequalities in healthcare use. These reflect access to healthcare, which is shaped on a smaller scale than the national level, by the institutions and establishments of a health system that individuals interact with on a regular basis. This scoping review maps the existing evidence about the influence of mesolevel access characteristics and socioeconomic position on healthcare use. Furthermore, it summarizes the evidence on the interaction between mesolevel access characteristics and socioeconomic inequalities in healthcare use.

**Methods:**

We used the databases MEDLINE (PubMed), Web of Science, Scopus, and PsycINFO and followed the ‘Preferred Reporting Items for Systematic Review and Meta-Analysis Protocols extension for scoping reviews (PRISMA-ScR)’ recommendations. The included quantitative studies used a measure of socioeconomic position, a mesolevel access characteristic, and a measure of individual healthcare utilisation. Studies published between 2000 and 2020 in high income countries were considered.

**Results:**

Of the 9501 potentially eligible manuscripts, 158 studies were included after a two-stage screening process. The included studies contained a wide spectrum of outcomes and were thus summarised to the overarching categories: use of preventive services, use of curative services, and potentially avoidable service use. Exemplary outcomes were screening uptake, physician visits and avoidable hospitalisations. Access variables included healthcare system characteristics such as physician density or distance to physician. The effects of socioeconomic position on healthcare use as well as of mesolevel access characteristics were investigated by most studies. The results show that socioeconomic and access factors play a crucial role in healthcare use. However, the interaction between socioeconomic position and mesolevel access characteristics is addressed in only few studies.

**Conclusions:**

Socioeconomic position and mesolevel access characteristics are important when examining variation in healthcare use. Additionally, studies provide initial evidence that moderation effects exist between the two factors, although research on this topic is sparse. Further research is needed to investigate whether adapting access characteristics at the mesolevel can reduce socioeconomic inequity in health care use.

**Supplementary Information:**

The online version contains supplementary material available at 10.1186/s12939-024-02122-6.

## Background

Individuals in socially disadvantaged situations often experience higher levels of morbidity and mortality [1]. Variations in health outcomes may result from differences in the use of healthcare. Equity in the distribution of healthcare is therefore a goal of many health systems [2]. Thus, the design and management of health systems are crucial in achieving health equity [3, 4]. A large body of research has examined health system structures and elements that address health equity. This research underlines the potential and responsibility of health systems to contribute to the achievement of health equity [3, 5, 6]. A key element in that context is access to healthcare facilities. Equality of access is the prerequisite for health equity [7–12]. In the assessment of equality in access and the role of health systems in this context, healthcare use plays a key role [13]. According to Andersen [13], healthcare use can be seen as a measure of realised ‘effective access’, and is a commonly used measure to represent access and socio-economic differences in access [14–19].

Health system characteristics and their contribution to equality in access often focus on macrolevel characteristics that are typically defined by national legislation [20–22]. These comprise measures such as the resources spent for healthcare facilities [23], national expenditure levels [24], the extent of co-payments, or the presence of gate-keeping systems [22].

**Fig. 1:**
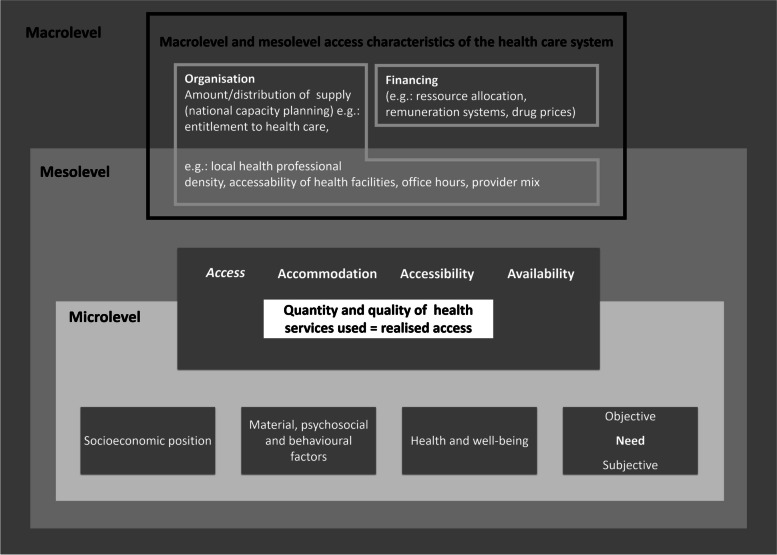
A framework to distinguish the macro- and mesolevel and their influence on access and utilisation of health care services

 Even though many high-income countries already perform well on these indicators, inequities in healthcare use and health outcomes remain evident [15, 25–27]. While most research so far has focused on macroeconomic level policies at a national level, the question arises whether there might be potential to improve equity in health and access to health services on a smaller scale. We refer to this smaller level as the ‘mesolevel’ as it lies below the macrolevel, yet also differs from the micro (individual) level characterized by the personal characteristics of individual health care users [28, 29]. Aday and Anderson (1974) refer to the microlevel as characteristics of the population at risk [30]. These three levels are partially intertwined: We for instance observe that the organisation at the macrolevel in financing and capacity planning in a country greatly influences the decisions on the mesolevel regarding the local density of physicians in a region. Furthermore, we encounter a certain overlap between the micro- and mesolevel. The interaction between the individual and representatives of the health care system is characterised by a series of one-to-one relationships between health care providers and patients. While each of these contacts takes place at an individual level, i.e. at the microlevel, the collective of these contacts can be regarded as being part of a mesolevel. The structure of these levels and their overlaps are depicted in Fig. 1.

Various definitions of the mesolevel can be found in the literature [28, 31, 32]. In particular, the mesolevel describes characteristics of health systems on a scale smaller than the national level. It focuses attention on factors and responsibilities at sub-national levels – often geographic regions such as counties or districts – and refers to them as ‘local’ or ‘regional characteristics.’

Kramer et al. [28] define the mesolevel of the health system ‘as the institutions and establishments that individuals interact with on a regular basis.’ According to this view, it is the local design of health services and the structure of the supply side that determines the interaction between patients and the health system. Characteristics of a health system’s mesolevel might influence the use of health services based on access to healthcare, in terms of ‘accommodation’ (e.g. office hours), ‘accessibility’ (e.g. travel time) and ‘availability’ (e.g. regional physician density) on the individual level, as defined by Penchansky and Thomas [10] (depicted in Fig. 1). Therefore, also the design of the health system at the mesolevel should not be overlooked when examining inequalities in health and healthcare use.

A vast of quantitative studies on mesolevel characteristics of health systems and how they influence socioeconomic inequalities in healthcare use exists. However, a comprehensive review of this evidence is still missing. We therefore aim to assess the extent of available evidence on the effects of mesolevel access characteristics of health systems and socioeconomic position (SEP) on healthcare use. Additionally, the relationship between mesolevel access characteristics and SEP shall be investigated and gaps in the body of evidence will be identified. Therefore, this scoping review aims to answer the following research question:

*Which mesolevel access characteristics of the health system and socioeconomic factors influence healthcare use and how do access characteristics interact with socioeconomic inequalities in healthcare use?*

We consider research that examines the influence of mesolevel access characteristics of the health system and SEP on healthcare use. We will then assess which research considers the interaction between mesolevel access characteristics and SEP in healthcare use.

## Methods

This scoping review was conducted according to guidance provided by the ‘Preferred Reporting Items for Systematic Review and Meta-Analysis Protocols extension for scoping reviews (PRISMA-ScR)’ [33] and the Joanna Briggs Institute [34]. A protocol of this scoping review describing the approach in detail was published in advance [35].

### Inclusion criteria

To be eligible for inclusion, a study had to meet the following three criteria:

1. Measure of individual healthcare use must be reported.
2. Mesolevel access characteristic of the health system must be included in the analysis.
3. Measure of SEP must be included in the analysis.

A detailed description of these criteria is given in Table 1 and in the following subsections:

#### Participants

We did not restrict our literature search on participants of a certain age, gender or morbidity. The decision to include all populations follows the rationale that access characteristics showing correlations with SEP and healthcare use at the mesolevel for any type of patient could also be relevant in specific age groups such as children and adolescents. However, due to the specific aim of the project (Understanding inequity in the healthcare use in children and adolescents) the scoping review was conducted for, the number of studies focussing on children and adolescents will be explicitly mentioned.

#### Outcomes

Outcome measures include various measures of healthcare use such as the number of physician visits or hospitalisations. Studies that focused on outcomes unrelated to healthcare use, such as self-reported health or physical activity, were excluded.

#### Expositions of interest

The scoping review includes studies analysing mesolevel access characteristics associated to health services, e.g. traveling distance to the nearest physician. Studies that solely included macrolevel access characteristics such as insurance schemes or payment incentives were excluded. Furthermore, studies that investigated specific policy programmes, such as the effect of invitation letters on healthcare use, were excluded from our review as they do not reflect access to healthcare.

We included studies that investigate at least one SEP measure, e.g. income or education. Since the focus of this scoping review is socioeconomic *inequity* in healthcare use, we excluded studies that investigated populations of homogenous SEP. We also excluded studies that use ambiguous measures of SEP, such as rurality/urbanity, a measure that might indicate area level SEP, but also the health services’ supply structure. Finally, we excluded studies that have a focus on the cultural contexts (e.g. language barriers or cultural beliefs) as these factors are beyond the scope of this review.

#### Study types

We included various quantitative study designs on human populations (e.g. cross-sectional studies, prospective studies, cohort studies, case-control studies). We only considered original and peer-reviewed research articles while comments, letters, and statements were excluded. In comparison to the study protocol we refrained from the inclusion of qualitative studies due to its high heterogeneity in analyses topics.

Aiming to increase the comparability and transferability of our findings across countries, we only considered studies from high-income countries (categorised as ‘developed economies’ in the classification of the United Nations [36]), as it is plausible to assume that health systems, access to healthcare, and socioeconomic disparities differ significantly between high-, middle and low-income countries.

The search was restricted to articles written in English or German published between 01.01.2000 and 31.03.2020. Observation periods of the studies must also be in that time frame.

**Table 1:** Overview of inclusion and exclusion criteria

	Inclusion/Exclusion
Study designs	Original and peer-reviewed quantitative research articles
Population	No restriction
Country	High-income countries according to the UN classification
Determinants of interest	1. A measure of socioeconomic position, e.g. • educational attainment • income • deprivation • occupational status (Excluding studies that focussed on groups with one homogenous socioeconomic position)
	2. Mesolevel access characteristics, e.g.: • physician density at a regional level • distance to physician • travel time to physician • office hours (Excluding determinants of access at the macrolevel, e.g.: insurance status, provider payment schemes)
Outcomes	Any measure of individual healthcare use, e.g. • physician visit • hospital visit (Excluding studies that focussed on health status or health-related behaviour, e.g.: physical activity, smoking)
Languages	German, English
Publication date, data basis	01.01.2000-31.03.2020

### Search strategy

We used the databases MEDLINE (PubMed), Web of Science, Scopus and PsycINFO. The search strategy considered three thematic blocks of keywords that reflect the main inclusion criteria. The blocks were connected with a Boolean operator AND ‘Healthcare use’ AND ‘mesolevel access characteristics’ AND ‘SEP measures’. The defined keywords were applied to a search within titles and abstracts. If applicable, appropriate MeSH terms were also searched. Furthermore, language and publication dates were operationalized in the search strategy.

Although this scoping review is restricted to studies conducted in high-income countries, this was not explicitly reflected in the search term, but filtered afterwards. The full search strategy, including the applied search terms for each database, is available in supplementary Table 1.

### Study selection process

The identified articles were combined and de-duplicated using EndNote software. The selection process consisted of two screening stages. First, two reviewers working independently screened titles and abstracts according to the predefined inclusion criteria using the software Rayyan [37]. In the second stage, three reviewers working independently conducted a full-text review. The inter-rater agreement between the reviewers was assessed by calculating Cohen’s Kappa of each phase of the selection process. Disagreements were resolved by discussions among the reviewers.

### Data extraction

A standardized data extraction form was developed in advance to the extraction process. The information extracted from the full-text articles were: author name, year of publication, name of the study, aim of the study, study country, observed study period, study population (age, disease focus), sample size, study design, statistical methods/analysis, healthcare use measure(s), measure(s) of SEP, access characteristic(s) analysed, control variables in the analyses, and main findings.

One reviewer performed the data extraction. 30% of studies were double extracted by a second reviewer to ensure accuracy of data extraction.

Critical appraisal of evidence quality is usually not provided in scoping reviews, and was not performed for this review [38].

### Data synthesis

Data synthesis was performed in three steps. First, outcomes were grouped into inductively derived categories. Three researchers developed and refined these in an interactive process. All included studies were subsequently summarized in a table using the categories derived. Second, the quantitative study results were summarized graphically. Finally, all results were used to narratively synthesize the evidence.

### Patient and public involvement

No patients involved.

## Results

### Search and selection of included studies

**Fig. 2:**
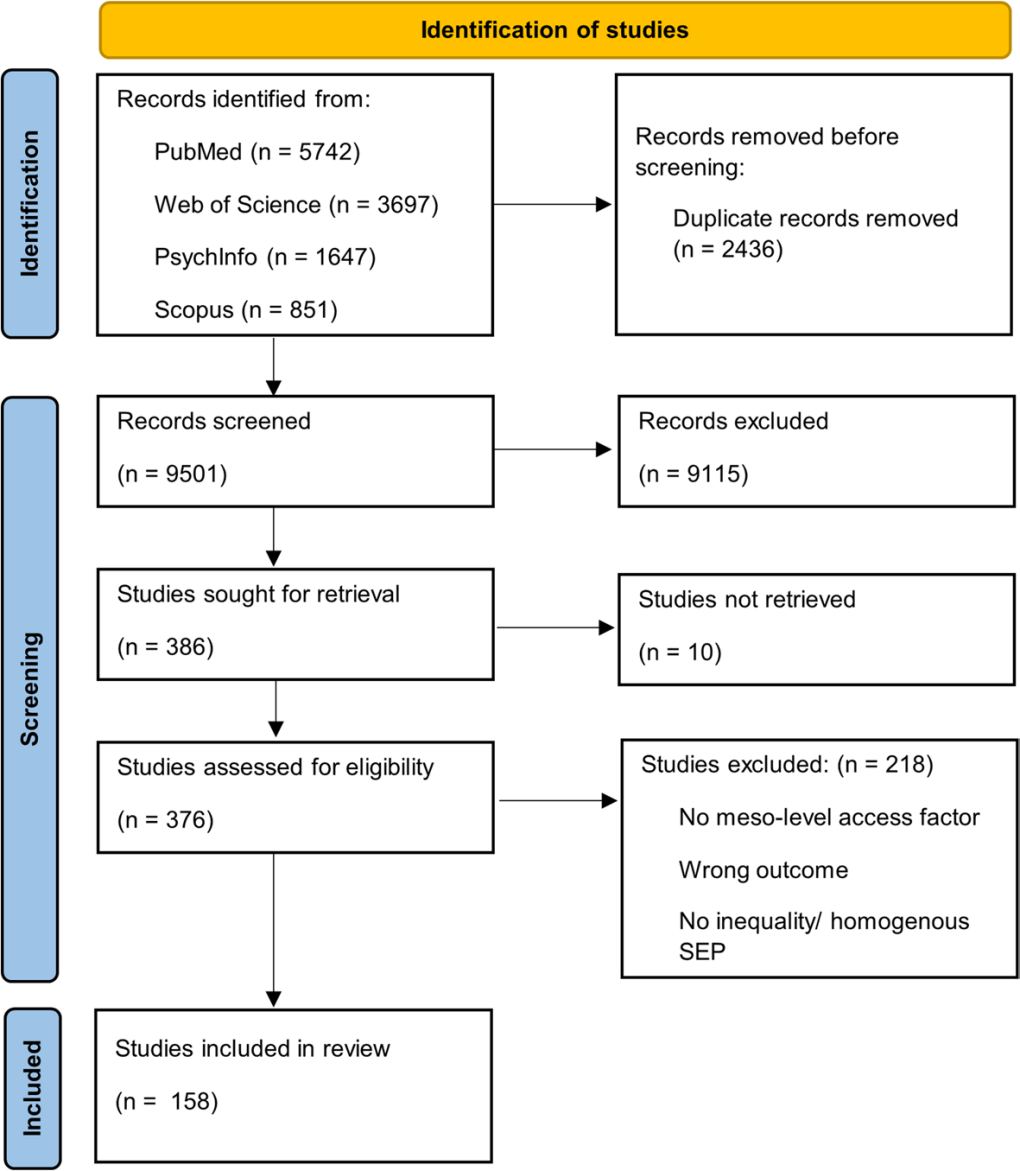
PRISMA flow diagram of study selection process [39]

A total of 11,937 articles were identified, from which 2,436 records were removed as duplicates. This resulted in 9,501 records, screened in the first stage. 386 studies passed the first screening stage with a high inter-rater agreement (Cohen’s Kappa = 0.89). 376 studies could be retrieved and were assessed in full-text screening. From these, 217 studies were excluded. Main exclusion reasons were missing access or SEP variables and inadequate outcomes. The second screening phase also resulted in a Cohen`s Kappa of 0.89. A total of 158 articles were included in the scoping review. The selection of studies is depicted in Fig. 2.

### Characteristics of included studies

In total, 158 studies from 18 countries were included in the scoping review. Table 2 lists all the studies and their main characteristics. A detailed list with further study characteristics can be found in the Appendix. Most of the studies originate from the United States (*n* = 72), followed by Canada (*n* = 18), UK (*n* = 14), and France (*n* = 11). All but one studies were written in English (*n* = 157), except one in German.

The included studies investigated socioeconomic differences in healthcare use of children (*n* = 21), adults (*n* = 85), elderly (*n* = 19), or a general population (*n* = 33). Disease-specific analyses focussed on healthcare use of patients with diabetes, asthma, cancer, mental illnesses, myocardial infarction, heart failure, or pneumonia. Some further studies investigated healthcare-seeking behaviour in cases of pregnancies, natal care, and recipients of hip joint replacements, knee joint replacements, or transplants. We describe our results based on 1,339 correlations derived from quantitative studies.

The results of the scoping review indicate limited evidence regarding the interaction of mesolevel access characteristics and socioeconomic inequality in healthcare use. Most of the studies consider the effect of SEP on healthcare use or the impact of access factors on healthcare use, but only a small number of studies investigate interaction of effects.

Outcomes were inductively categorised into three types by the authors based on the included studies: the use of curative, preventive, and potentially avoidable services. The categories shall reflect the different meanings of the outcome variables and their interpretation. While preventive service use includes preventive services for specific diseases or irrespective of a disease, curative use comprises all services demanded as a consequence of a disease or for unspecified reasons. Potentially avoidable service use subsumes services that may be perceived as negative - i.e. those for which a high claim reflects a high disease burden. One example of potentially avoidable service use is an avoidable hospitalisation, i.e. one that might have been avoided through earlier, more adequate care. Further, the category of potentially avoidable use includes studies where outcomes reflected unmet needs. Studies that used multiple outcomes referring to different types of use are categorised as mixed outcomes in Table 2.

A total of 28 studies investigated use of preventive services. These included outcomes such as doctor visits for preventive counselling, screenings, vaccinations, eye examinations, and dental check-ups. Fifty-six studies focussed on curative services use such as GP/specialist visits, hip/knee replacements, complementary or alternative medicine use. Fifty-nine studies examined potentially avoidable outcomes including unplanned ED visits, avoidable hospitalisations, and amputations. Fifteen studies investigated outcomes of more than one category. Of the 21 studies that focussed on children or adolescents, 3 investigated socioeconomic differences in the use of preventive services such as dental care use and vaccinations. Nine studies focussed on curative service use with focus on outcomes such as the number of healthcare visits, and eleven studies considered potentially avoidable healthcare use in ED visits and (avoidable) hospitalisations.

The most frequently used indicator of the SEP was income (*n* = 84 studies), followed by measures of education (*n* = 81 studies). Further measures of SEP were poverty or deprivation (*n* = 53), marriage and family structure (*n* = 43), employment status (*n* = 39), migration (*n* = 12) or composite indexes (*n* = 15).

Mesolevel access characteristics included the accessibility of primary care (general practitioners), secondary care (specialists), and tertiary care (hospitals), the density of providers, distance to providers, and driving time. Further access factors investigated were the ownership and volume of hospitals, teaching status, and office hours.

**Table 2:** Characteristics of the included studies

Outcome category	Author (Year)	Country	Study population	Outcome (Assessment method)	Focus on moderation between SEP and access
Use preventive services	Meersman et al. (2009) [40]	USA	adults (40–84 years, women)	mammography screening use (survey)	
	Mobley et al. (2009) [41]	USA	adults (65–104 years, women)	mammography screening use (administrative data)	
	Haas et al. (2010) [42]	USA	adults (≥ 50 years)	colorectal cancer screening use (survey)	
	Patel et al. (2010) [43]	USA	adults (≥ 45 years)	prostate cancer screening use (survey)	
	Smith et al. (2011) [44]	USA	adults (≥ 41 years, women)	mammography screening use (survey)	
	Patel et al. (2012) [45]	USA	adults (≥ 50 years)	colorectal cancer screening use (survey)	
	Akinyemiju et al. (2012) [46]	USA	adults (50–74 years, women)	mammography screening/clinical breast examination use (survey)	
	McCall-Hosenfeld et al. (2012) [47]	USA	adults (18–45 years)	screening and vaccination index; preventive counselling index (survey)	
	Jensen et al. (2014) [48]	Denmark	adults (50–70 years, women)	mammography screening use (administrative data)	
	Charland et al. (2014) [49]	Canada	population in Montreal	influenza A/H1N1p vaccination use (administrative data)	
	Luo et al. (2014) [50]	USA	adults (≥ 18 years, with diabetes)	influenza and pneumococcal vaccination use; doctor’s visit; A1C test use; foot and eye examination; self-care education (survey)	
	Marino et al. 2014 [51]	Australia	adults (≥ 55 years)	dental care use (time interval since last dental visit; survey)	
	Ouedraogo et al. (2014) [52]	France	adults (51–74 years, women)	mammography screening use (administrative data)	
	Patel et al. (2014) [53]	USA	adults (≥ 40 years, women)	mammography screening/clinical breast examination use (survey)	
	Vogt et al. (2014) [54]	Germany	individuals in German districts	use of cancer screenings (prostate, cervical, colon, skin, mammography) (administrative data)	
	Henry et al. (2014) [55]	USA	adults (40–74 years, women)	mammography screening use (survey)	
	Dumas / Polk (2015) [56]	USA	children (15 months-5 years)	dentist visit (survey)	
	Sakai et al. (2015) [57]	Japan	children (< 15 years)	diphtheria, pertussis, tetanus, measles vaccination (administrative data)	
	Toivakka et al. (2015) [58]	Finland	adults (with type 2 diabetes)	hemoglobin A1c test use (patient file data)	
	Chou et al. (2016) [59]	USA	adults (≥ 40 years)	dilated eye examination use; eye care visits (survey)	
	Leinonen et al. (2017) [60]	Norway	adults (25–69 years, women)	cervical cancer screening use (administrative data)	
	Feng et al. (2017) [61]	USA	adults (≥ 18 years)	dental visits (county-level percent of visits in the previous year) (survey)	
	Fujita et al. (2017) [62]	Japan	adults (40–74 years)	use of annual health check-up (administrative data)	yes
	Héquet / Rouzier (2017) [63]	France	children (10–19 years, females)	HPV vaccination rate (administrative data)	
	Jewett et al. (2018) [64]	USA	adults (50–74 years, women)	mammography screening use frequency (survey)	yes
	Yoon et al. (2018) [65]	USA	adults (≥ 18 years)	use of preventive dental care (survey)	
	Wright et al. (2019) [66]	UK	adults (≥ 60 years)	attendance at publicly funded eye examination (census data)	
	Patel et al. (2020) [67]	USA	adults (≥ 40 years, women)	cervical cancer screening/mammography/colorectal cancer screening use (survey)	
Potentially avoidable use	Kirby / Kaneda (2005) [68]	USA	adults (≥ 18 years, women)	unmet medical need (inability to obtain health care when participant thought it was necessary) (survey)	
	Kirby / Kaneda (2006) [69]	USA	adults (> 25 years)	poor access to healthcare (survey)	
	Giorda et al. (2006) [70]	Italy	adults (20–75 years)	ED visits; re-admissions for diabetes-related complications; unplanned hospital admissions (hospital data)	
	Ionescu-Ittu et al. (2007) [71]	Canada	adults (elderly, ≥ 65 years)	rate of ED use (administrative data)	
	Harris et al. (2008) [72]	USA	population	myocardial infarct hospitalisations; heart failure hospitalisations (hospital data)	
	Penfold et al. (2008) [73]	USA	children (2–20 years)	risk of perforated appendicitis (hospital data)	
	Chen et al. (2009) [74]	USA	adults	hospitalisations due to ambulatory care sensitive conditions (administrative data)	
	Knudson et al. (2009) [75]	USA	children (2–17 years)	hospitalisations for asthma (hospital data)	
	Concannon et al. (2009) [76]	USA	adults (≥ 18 years)	elapsed time in emergency medical services; delay in emergency medical services (hospital data)	
	Rosato et al. (2009) [77]	Italy	adults (incident breast cancer patients)	breast-conversing therapy surgery with/without radiotherapy/mastectomy use (administrative data)	
	Margolis et al. (2011) [78]	USA	children, adults (full population of U.S. Medicare beneficiaries with diabetes)	lower-extremity amputation incidence (administrative data)	
	Magán et al. (2011) [79]	Spain	adults (≥ 65 years)	hospitalisations due to ambulatory care sensitive conditions (hospital data)	
	Pracht et al. (2011) [80]	USA	individuals in counties	rate of avoidable hospitalisations (hospital data)	
	Hsia et al. (2011) [81]	USA	individuals treated in hospitals	proportion of patients leaving the ED without being seen (administrative data)	
	Grillo et al. (2012) [82]	France	adults (≥ 18 years, women)	absence of cervical cancer screening (survey)	
	Borda-Olivas et al. (2013) [83]	Spain	adults (> 65 years)	rate of avoidable hospitalisations (hospital data)	
	Butler et al. (2013) [84]	Australia	children (0–4 years)	hospitalisations due to ambulatory care sensitive conditions (administrative data)	
	Cavalieri (2013) [85]	Italy	adults (≥ 18 years)	having a self-reported unmet medical need (survey)	yes
	Harrington et al. (2013) [86]	Canada	children, adults (> 12 years)	reporting difficulty accessing specialist care (survey)	yes
	Rudge et al. (2013) [87]	UK	children (5–15 years), adults (≥ 15 years)	ED visits (administrative data)	yes
	Tao et al. (2013) [88]	Canada	individuals in 47 major cities/towns in Ontario	cardiac surgery use (administrative data)	
	Willems et al. (2013) [89]	Belgium	children, adults	use of out of hours care in ED rather than primary care (patient records)	
	Mathison et al. (2013) [90]	USA	children (0–13 years)	non-urgent ED visits (administrative data)	
	Blain et al. (2014) [91]	UK	children (0–14 years)	hospitalisations for pneumonia (administrative data)	
	Basu / Mobley (2014) [92]	USA	adults (≥ 65 years)	hospitalisations due to ambulatory care sensitive conditions (hospital data)	
	Kottwitz (2014) [93]	Germany	children (newborns)	likelihood for caesarean section (survey)	yes
	Hunold et al. (2014) [94]	USA	adults (≥ 65 years)	ED visits (administrative data)	
	White et al. (2014) [95]	UK	individuals with mental illnesses	rates of hospital admissions for severe mental illness (administrative data)	
	Herrin et al. (2015) [96]	USA	adults (discharged with myocardial infarction, heart failure, or pneumonia)	hospital readmission rates (administrative data)	
	Mercier et al. (2015) [97]	France	population	rate of avoidable hospitalisations (administrative data)	
Potentially avoidable use	Slaunwhite (2015) [98]	Canada	children, adults (≥ 15 years)	self-reported barriers to mental healthcare (survey)	yes
	Fisher-Owens et al. (2016) [99]	USA	children (2–17 years)	absence of a preventive dental visit (survey)	
	Lee et al. (2016) [100]	USA	individuals in census tracts	ED visits (administrative data)	
	Fusco et al. (2016) [101]	Italy	adults (≥ 18 years)	avoidable hospitalisations (administrative data)	
	Sheringham et al. (2017) [102]	UK	individuals in administrative areas	hospitalisations due to ambulatory care sensitive conditions (administrative data)	
	Chalmers (2017) [103]	USA	population of Maryland	ED discharges for dental/oral conditions (administrative data)	
	Lines et al. (2017) [104]	USA	children, adults (enrollees with commercial insurance)	ED visits; ED visits due to ambulatory care sensitive conditions (administrative data)	
	Noah (2017) [105]	USA	adults (women who had a live birth in 2008)	inadequate use of prenatal care index (administrative data)	
	Alcala et al. (2018) [106]	USA	children (0–14 years)	avoidable asthma related hospitalisation; preventable asthma related ED visits (hospital data)	
	Fishman et al. (2018) [107]	USA	adults (18–87 years)	ED visits for preventable conditions (hospital data)	
	Schmidt et al. (2018) [108]	USA	adults (≥ 18 years)	avoidable ED visits, avoidable hospitalisations (administrative data)	
	Collins et al. (2018) [109]	Australia	adults (women with breast cancer)	mastectomy rates vs. breast conserving surgery (administrative data)	
	Maeda et al. (2018) [110]	Japan	adults (women)	caesarean section rates (administrative data)	
	Carmeiro (2018) [111]	Portugal	individuals with hospitalisations in NHS hospitals	hospitalisations due to ambulatory care sensitive conditions (administrative data)	
	Delgadillo et al. (2018) [112]	UK	individuals in 144 IAPT providers that covered 180 local areas	percentage of cases that did not receive psychological treatment (administrative data)	
	Lavoie et al. (2018) [113]	Canada	individuals of First Nations living both on and off reserve	hospitalisations for mental health related ambulatory care sensitive conditions (administrative data)	
	Or / Penneau (2018) [114]	France	adults (≥ 65 years)	ED visits (administrative data)	
	Stracci et al. (2018) [115]	Italy	children, adults (< 80 years)	delivery of radiotherapy (administrative data)	
	Gartner et al. (2018) [116]	USA	adults (18–44 years)	hysterectomy rates (administrative data)	
	Daly et al. (2018) [117]	USA	adults (≥ 65 years)	avoidable hospitalisations (administrative data)	
	Shoff et al. (2019) [118]	USA	individuals in U.S. counties	ED admissions (administrative data)	
	Ranade et al. (2019) [119]	USA	adults (> 18 years)	ED visits for non-traumatic dental conditions (administrative data)	
	Roy et al. (2019) [120]	USA	children, adults	all cause hospitalisation rates (administrative data)	
	Jayasekera et al. (2019) [121]	USA	adults (≥ 70 years)	advanced prostate cancer diagnosis (administrative data)	
	Coyle et al. (2019) [122]	UK	adults (≥ 18 years)	prevalence of uncontrolled hypertension (administrative data)	
	Okuyama et al. (2019) [123]	Japan	children, adults (> 15 years)	risk of untreated hypertension (administrative data)	
	Renner (2020) [124]	Austria	individuals in Austrian districts	hospitalisations for acute and ambulatory care sensitive conditions (administrative data)	
	Carruth et al. (2006) [125]	USA	adults (≥ 18 years, women)	failure to obtain cervical cancer screening (survey)	
Curative service use	Maheswaran et al. (2003) [126]	UK	children, adults (15–84 years)	renal replacement therapy rates (haemodialysis; peritoneal dialysis; transplantation) (hospital data)	
	Woods et al. (2003) [127]	USA	children (< 5 years; ≥5 years)	number of healthcare visits (survey)	
	Vanasse et al. (2005) [128]	Canada	adults (women / men, ≥ 65 years)	bone mineral density test use (administrative data)	
	Chaix et al. (2005) [129]	France	adults (> 65 years)	specialists visits in relation to PCP visits (survey)	
	Field / Briggs (2001) [130]	UK	children, adults (diabetics, asthmatics)	frequency of primary care use (survey)	
	Cadarette et al. (2007) [131]	Canada	adults (65–89 years)	DXA testing; treatment (alendronate, etidronate, risedronate, calcitonin, and/or raloxifene) (survey)	
	Judge et al. (2009) [132]	UK	adults (≥ 50 years)	hip/knee replacement rates (administrative data)	
	Magner et al. (2009) [133]	USA	medicaid enrollees	carotid endarterectomy use (administrative data)	
	Gage et al. (2009) [134]	UK	adults (cancer patients)	complementary and alternative medicine use (survey)	
	Tonner et al. (2010) [135]	USA	adults (≥ 18 years)	self-reported number of physician visits for systemic lupus erythematosus (survey)	
	Diaz-Granados et al. (2010) [136]	Canada	children, adults (≥ 15 years)	use of general practitioner/family physician services for mental healthcare; psychiatric services for mental health reasons (survey)	
	Barner et al. (2010) [137]	USA	adults (≥ 18 years)	complementary and alternative medicine use (survey)	
	Rubin et al. (2011) [138]	Denmark	adults (40–90 years)	use of DXA scanning (administrative data)	
	Bronstein et al. (2011) [139]	USA	adults (mothers covered by Arkansas Medicaid)	likelihood of infant delivery at NICU (administrative data)	
	Haroon et al. (2011) [140]	UK	children, adults (residents who received an antiviral drug for influenza-like illness)	antiviral collection rates (administrative data)	
	Telleen et al. (2012) [141]	USA	children (4–8 years)	frequency of dental visits; continuity of care; initiation of care (survey)	
	Judge et al. (2012) [142]	UK	adults (≥ 20 years)	rates of renal replacement therapy (administrative data)	
	Ryvicker et al. (2012) [143]	USA	adults (60–99 years)	primary care visit use in past 12 months (survey)	yes
	Goswami et al. (2012) [144]	USA	adults (> 17 years)	latent tuberculosis infection treatment initiation/completion (survey)	
	Harrington et al. (2012) [145]	Canada	adults	realized access to PCPs (survey)	
	Archibald / Rankin (2013) [146]	USA	individuals in U.S. counties	substance abuse disorder assessment, assessment of other mental health problems (survey)	
	Cook et al. (2013) [147]	USA	adults (≥ 18 years)	mental health service use (survey)	
	Schäfer et al. (2013) [148]	Germany	individuals in German counties	rates of hip/knee replacement (administrative data)	
	Bocquier et al. (2013) [149]	France	adults (18–64 years)	new/long antidepressant treatment (administrative data)	
	Lemstra et al. (2013) [150]	Canada	adults (with ischemic heart disease)	cardiac rehabilitation attendance (exercise component; completion) (administrative data)	yes
	Neri et al. (2013) [151]	Italy	adults (≥ 18 years)	transplant waiting list activation (administrative data)	
	Yasaitis et al. (2013) [152]	USA	medicare beneficiaries, cardiologists, and primary care physicians	outpatient visit rates (administrative data)	
	Hadlock et al. (2013) [153]	Canada	adults (≥ 18 years)	open-access colonoscopy use (administrative data)	
	Kopetsch / Schmitz (2014) [154]	Germany	individuals in German counties	number of GP/specialist/ physiotherapist consultations(administrative data)	
	Chamberlain et al. (2014) [155]	USA	children (0–18 years)	inpatient use of pediatric cancer specialty centers (administrative data)	
	Huang et al. (2014) [156]	USA	children, adults	use of high-volume hospitals for colorectal cancer (administrative data)	
	Ozegowski / Sundmacher (2014) [157]	Germany	individuals in German districts	equity index (degree of disparity between need for and actual use of outpatient health services) (administrative data)	
	Widdifield et al. (2014) [158]	Canada	adults (newly diagnosed patients with RA)	percentage of patients with incident rheumatoid arthritis (RA) with rheumatologist visit (administrative data)	
	Alruwaily et al. (2015) [159]	USA	adults (> 17 years)	follow-up testing for nephrolithiasis (administrative data)	
	Annequin et al. (2015) [160]	France	adults (30–79 years)	reimbursement of antidepressants, private psychiatrist visits (administrative data)	
	Badley et al. (2015) [161]	Canada	adults (> 18 years)	physician visits for arthritis (survey)	
	Pasnisinova et al. (2016) [162]	Czech Republic	adults (patients underwent heart transplantation)	Incidence of heart transplantation (administrative data)	
	Chew et al. (2016) [163]	Australia	individuals in 61 Medicare locals	coronary angiography rate (administrative data)	
	Doumouras et al. (2016) [164]	Canada	adults (≥ 18 years)	rates of bariatric surgery (hospital data)	
	Okafor et al. (2016) [165]	USA	patients with inpatient colonoscopies	inpatient colorectal stent use (hospital data)	
	Alvarez et al. (2017) [166]	USA	children (0–18 years)	use of pediatric cancer specialty center (administrative data)	
	Kelly et al. (2017) [167]	UK	adults (women, born in Bradford cohort)	GP consultation rates (patient records)	
	Doumouras et al. (2017) [168]	Canada	adults (≥ 18 years)	Rates of bariatric surgery (administrative data)	
	Finley et al. (2017) [169]	USA	adults (veterans)	use of post-traumatic stress disorder care (administrative data)	
	Jabo et al. (2017) [170]	USA	children, adults (≥ 15 years)	cancer-directed chemotherapy status; hematopoietic cell transplantation (administrative data)	
	Rommel / Kroll (2017) [171]	Germany	adults (18–79 years)	physical therapy use (survey)	
	Ruhnke et al. (2017) [172]	USA	individuals (enrolled with a lower gastrointestinal bleeding)	esophagogastroduodenoscopy use (administrative data)	
	Cook et al. (2017) [173]	USA	adults (≥ 18 years)	initiation of mental healthcare; number of days of treatment during episode among those receiving any mental health treatment (survey)	
	Abbas et al. (2017) [174]	Germany	children (0–17 years)	nondrug psychiatric/psychotherapeutic treatment use (administrative data)	
	Walsh et al. (2017) [175]	USA	children (0–17 years)	lingual frenotomy use (hospital data)	
	Greiner et al. (2018) [176]	Germany	adults (> 17 years)	doctor visits (survey)	
	Johansson et al. (2018) [177]	Sweden	individuals in 21 Swedish regions (county councils)	primary care visits (administrative data)	
	Viana et al. (2018) [178]	Portugal	adults (≥ 18 years)	referral and completion of cardiac rehabilitation program (survey)	
	Régis et al. (2018) [179]	France	adults (≥ 17 years)	probability of breast reconstruction (administrative data)	
	van der Goes et al. (2019) [180]	USA	adults (18–100 years)	specialist physician visits for dementia/epilepsy/MS/Parkinson’s (administrative data)	
	Shah et al. (2019) [181]	USA	children (< 21 years)	rates of discharge with rehabilitative services (hospital data)	
Mixed service use	Sineshaw et al. (2020) [182]	USA	adults (≥ 35 years)	curative intent surgeries for early-stage non-small cell lung cancer (administrative data)	
	Arcury et al. (2005) [183]	USA	adults (> 18 years)	healthcare visits for regular check-ups; healthcare visits for chronic care; healthcare visits for acute care (survey)	
	Sørensen et al. (2009) [184]	Denmark	all inhabitants	referrals to outpatient hospital treatment; inpatient hospital treatment; referrals to private specialists (administrative data)	
	Petrelli et al. (2010) [185]	Italy	population	hospitalisations; out-patient care use; pharmaceutical care use (administrative data)	
	Guttmann et al. (2010) [186]	Canada	children (0–17 years)	hospitalisations due to ambulatory care sensitive conditions; ED visit; no preventative care visits; no primary care visits; no newborn visit; use for discretionary conditions (administrative data)	
	Zulian et al. (2011) [187]	Italy	adults (≥ 14 years)	mental health services use in hospitals; mental health services use (administrative data)	
	Sacerdote et al. (2012) [188]	Italy	adults (incident colorectal cancer patients)	postoperative in-hospital mortality; proportion of preoperative radiotherapy; proportion of abdominoperineal resection (hospital data)	
	Bielefeldt (2013) [189]	USA	individuals in U.S. states	admissions for gastroparesis; endoscopies; gastrostomies; nutritional support (administrative data)	
	Weeks et al. (2014) [190]	France	adults (45–99 years)	admissions for hip fracture; hip/knee replacement (hospital data)	
	Eibich / Ziebarth (2014) [191]	Germany	adults (17–100 years)	hospital use; ambulatory doctor visits (survey)	
	Gusmano et al. (2014) [192]	France	adults (> 20 years)	hospitalisations due to ambulatory care sensitive conditions; rates for revascularization – bypass surgery and angioplasty (administrative data)	
	Arnaout et al. (2015) [193]	Canada	adults (> 19 years)	mastectomy vs. no mastectomy; contralateral prophylactic mastectomy vs. no contralateral prophylactic mastectomy; preoperative breast MRI use (administrative data)	
	Posthumus et al. (2016) [194]	Netherlands	adults (> 20 years, singleton pregnancies)	labour in non-breech term and post-term pregnancies; referral during pregnancy from community midwife to obstetrician; elective caesarean section in term and post-term breech pregnancies; birth setting in low-risk pregnancies (administrative data)	
	Rowe et al. (2016) [195]	USA	adults (≥ 18 years)	opioid overdose deaths; overdose reversals (administrative data)	
	Klitkou et al. (2017) [196]	Norway	children (1–9 years)	hospital admissions; outpatient visits (administrative data)	
	Packness et al. (2017) [197]	Denmark	adults (20–64 years)	mental healthcare use (psych. emergency clinic; admissions);mental healthcare use (outpatient psychiatrist; psychologist; GP) (administrative data)	yes

### Results of the included studies

**Fig. 3:**
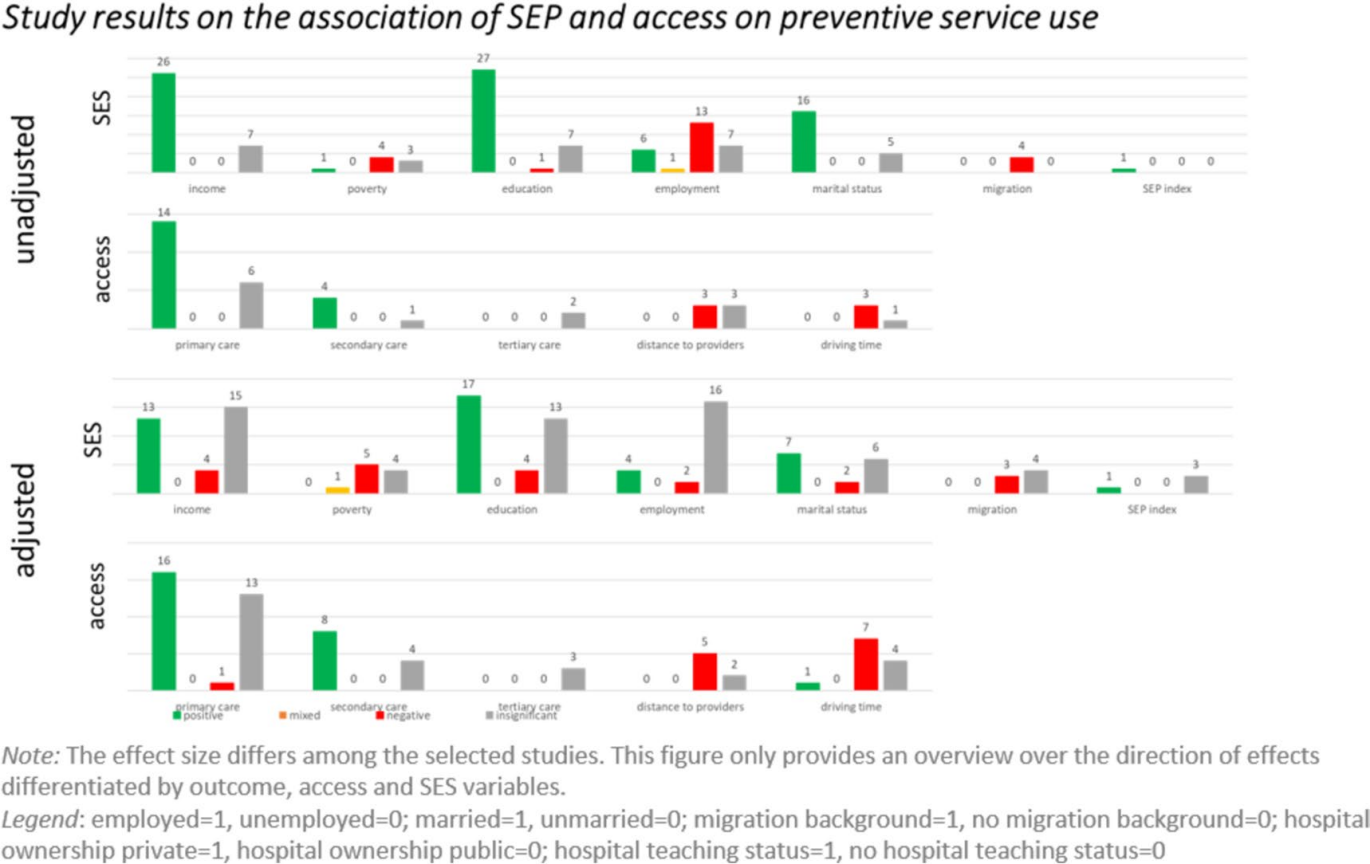
Harvest plots of the included study results on preventive service use

**Fig. 4:**
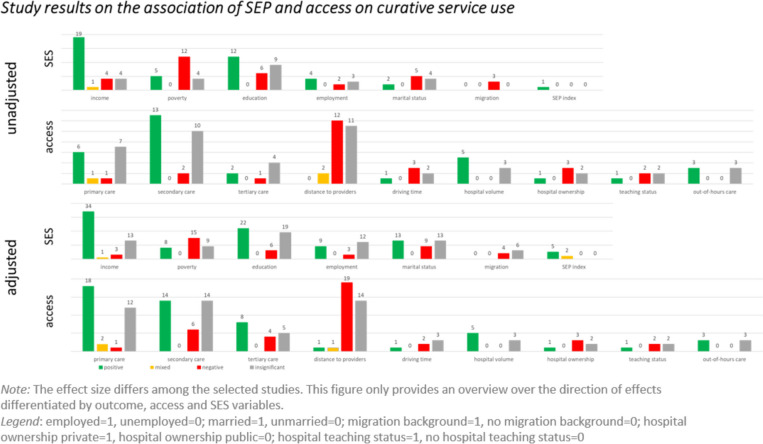
Harvest plots of the included study results on curative service use

**Fig. 5:**
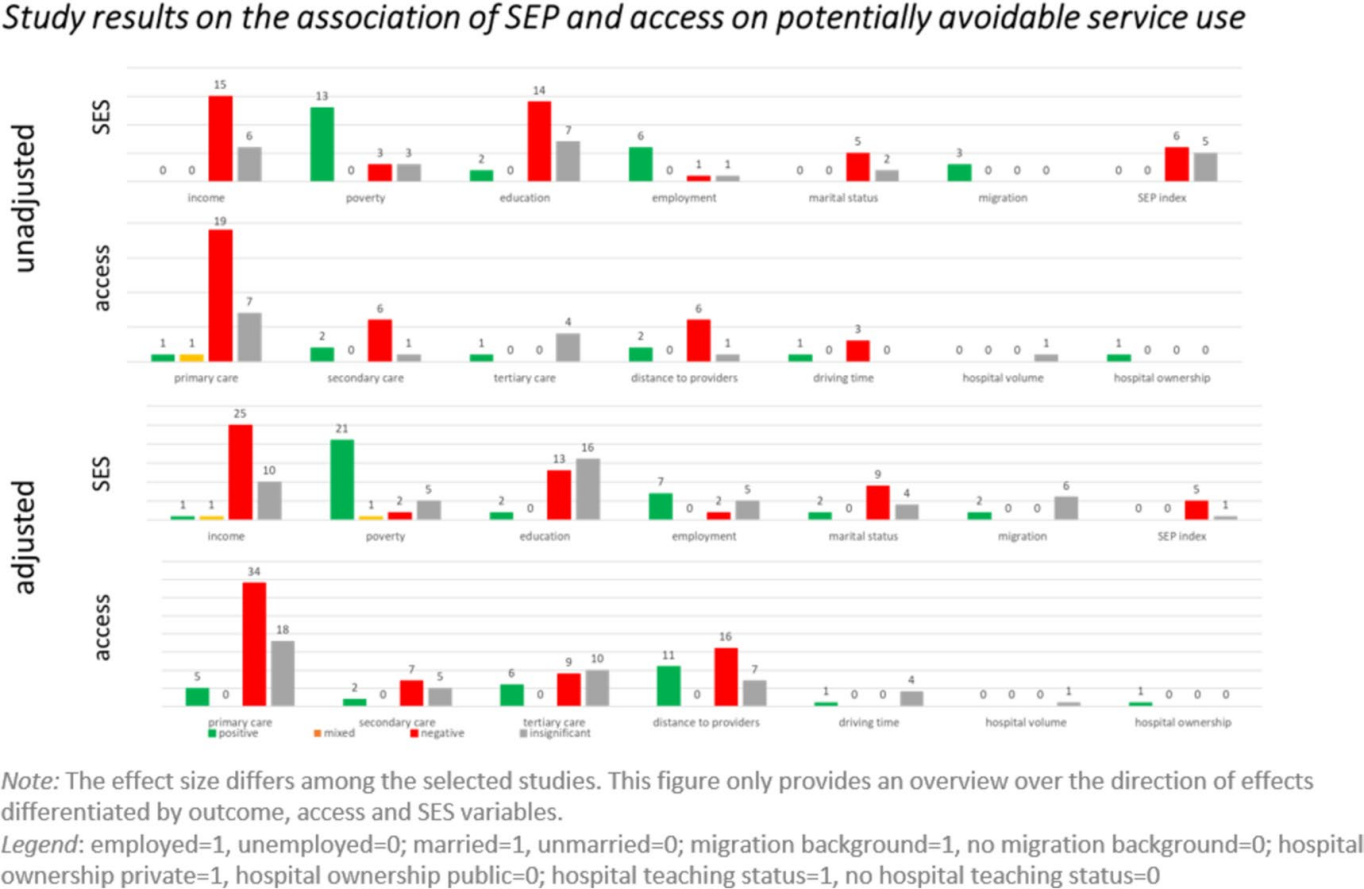
Harvest plots of the included study results on potentially avoidable service use

The results of 1,339 correlations are presented in modified harvest plots (Figs. 3, 4 and 5). These allow us to depict in a simplified manner tendencies of correlations despite the heterogeneity of the included studies. They consist of a set of bar charts for each of the three outcome categories. For each predictor, the number of correlations with a given conclusion on its effect on the respective outcome is represented by the bars’ height. The effect was evaluated as positive, negative, mixed, or insignificant. A ‘positive’ effect means that an increase of the respective predictor relates to an increase in healthcare use. A mixed effect is present when a predictor has a significant but non-monotonous effect on the outcome variable. Furthermore, we report unadjusted and adjusted correlations separately. Adjusted correlations refer to correlations resulting from statistical models that contain at least one SEP variable as well as at least one access variable. If statistical models included either only SEP variables or only access variables, the correlations were described as ‘unadjusted’.

Considering use of preventive services, some clear associations are visible. Income, education, and the availability of primary and specialist care are positively correlated with the use of preventive services. For example, 26 unadjusted correlations imply a positive influence of income as a predictor, 7 correlations were insignificant, and not a single negative correlation was found. Also, a strong association between marital status and use was derived. Sixteen unadjusted correlations show that married individuals are more likely to utilise preventive services, five correlations were insignificant, and no study showed negative correlations. Numerous studies investigate the influence of employment status on healthcare use across the three categories: the included studies demonstrate contradictory results and many insignificant correlations between both variables.

Most of the investigated access variables show the expected correlations, such as increasing distance to healthcare provider leading to decreased use curative services. However, this correlation is not as consistent in the category of potentially avoidable service use with 11 positive correlations, 16 negative, and 7 insignificant ones in adjusted models. One explanation might be that patients become more determined to overcome access barriers in emergency situations. In terms of physician availability, the amount of primary and secondary care facilities seems to have a stronger influence on healthcare use overall than the amount of tertiary care facilities.

### Results of studies reporting interaction effects

One objective of this scoping review was to analyse if studies investigated any interaction effect between mesolevel access characteristics and SEP in the context of healthcare use. Out of the 158 included studies, 10 investigated moderating effects. Among these, one study focussed on children. Nine out of ten studies reported that improved access had a significant effect on socioeconomic inequality in healthcare use. One study reported no evidence of effect modification. Six studies concluded that the higher an individual’s SEP, the less likely it is that barriers of distance and availability of healthcare providers will affect that person. In that context, SEP was defined either by income or by level of education. This means that the lower an individual’s SEP, the more susceptible that person is to increased distance to or lacking availability of healthcare services. Regarding the effect of accessibility on the effect of SEP on health care use, two studies reported that higher accessibility leads to education being a less significant predictor of healthcare. In contrast, another study reported that better accessibility increases use only for the better-off, but not for those in low-income neighbourhoods.

## Discussion

### Summary

Our scoping review shows that even though a good deal of research has been conducted on the influence of mesolevel access characteristics and socioeconomic differences on healthcare use, evidence about the interaction between these factors is still lacking. While we found 158 studies that met our inclusion criteria, only 10 of them considered how access factors and socioeconomic variables interact with each others effects on healthcare use. Nevertheless, 9 out of 10 studies reported significant interaction effects. Further research is needed to investigate the specificities of these.

Bringing together the identified correlations for the different use categories, we can see that the most unambiguous results appear regarding use of preventive services. A potential explanation is that this category comprises the most homogenous studies. Overall, most correlations follow the expected direction, and only a few unexpected results occurred. Most of the studies show the positive effects of increased income, education, and healthcare availability on use of preventive or curative services. To summarize, studies suggest that healthcare access and SEP serve as important factors for the use of preventive and curative services, such as cancer screenings, vaccination uptake, physician consultations, and antiviral collection rate.

The necessity of access-related efforts can have a negative effect on their use [134]. In contrast, the availability of healthcare facilities promotes use, in particular when distance and driving time are short. It follows that studies recommend services to be located near good transportation connections so that as many patients as possible can reach them [187]. Easy transportation might be especially important for vulnerable groups such as the elderly [138]. In this respect, the ability to reach health services without public transport of different populations must be considered [183]. Further characteristics of healthcare facilities such as clinic capacity can additionally influence use of services [49].

When interpreting these results thoughts should be given to the inverse-care law [198], which states that the availability of good medical care tends to correlate inversely with a populations’ need for services.

Concerning the influence of SEP related variables, the studies conclude that preventive and curative services are less used by socioeconomically deprived groups, irrespective of whether SEP is measured as individual income, individual education or area-level deprivation. This may consequently contribute to health disparities. As reasons for this phenomenon, studies list among other explanations, a possible lack of health literacy, and untailored communication strategies [140]. In contrast, patients with a higher SEP might be able to navigate through the healthcare system more efficiently [131]. Furthermore, depending on the healthcare system financial resources might be more or less necessary to devote to healthcare services, and thus pose a barrier to healthcare [45].

For potentially avoidable service use, such as avoidable hospitalisations most of the interrelations described above are reversing. The presented reasons are mostly identical to the other use categories, meaning that the lesser use of adequate preventive and curative services leads to higher potentially avoidable service use.

Other than that, being married appears to be a clear positive predictor for preventive service use, while being employed does not lead to clear positive effects.

The strong effect of marital status on healthcare use when comparing married to unmarried individuals is in line with the literature [199]. This relationship remains despite adjustment for potential confounders in multiple studies. The literature proposes several explanations: for instance, having a spouse or children might encourage people to feel more responsible for their own health, since the consequences of illness can affect family members. Another possibility is that a spouse advises his or her partner to use medical services when health problems arise. In both cases, health services might not have been used without the partner’s influence [199]. Furthermore, being married can increase individual´s time capacity to use healthcare services due to domestic divisions of labour and shared childcare [200].

Our results indicate an unclear relationship between employment status and healthcare use. Within the categories of curative and potentially avoidable use, more studies concluded that being unemployed increases the likelihood of using services. However, a high share of the included studies found an insignificant correlation. The scientific literature tends to see unemployment as an enforcing factor of healthcare use [201]. The main explanation for this correlation might be that unemployment is associated with health-related problems, and thus increased need for healthcare [202]. In contrast, employment may lead to time constrains and thus reduced health care use. Especially our results in the category of potentially avoidable service use, support this view. However, some studies find that being employed has a positive influence on use. A possible explanation could be that being employed increases financial resources, which depending on the health system might be necessary for access to healthcare. Additionally, employment in some countries is crucial for having insurance. Therefore, we conclude that the influence of employment on healthcare use must be investigated considering the financing of the health system, and adjusted for healthcare needs. Furthermore, it is crucial how and in which detail the variable is quantified. The type of employment is relevant [203], and also whether employment status was measured on an individual or a regional level. Many included studies used the share of unemployed individuals in an area. A high value of this variable might indicate an overall worse health status of the area’s population, and therefore increase use.

Despite that the included studies depict a broad variety of different variables which relate to access and SEP, not all existing barriers to healthcare are displayed in this review. In this respect qualitative studies are helpful to gather further information on hindering factors that might influence healthcare use. Especially, barriers that are not easily quantifiable such as the ability to get time off work or to find childcare can be illustrated in qualitative studies [45, 67].

### Limitations

Studies that investigate inequalities in healthcare use face the challenge of having to adjust for need factors. If no adjustment for need is present, it is hard to tell whether or not socioeconomic disparities cause the results. We sought to address this challenge by categorising the outcome measures into preventive, curative, and potentially avoidable service use. Yet due to the ambiguous nature of some health services, outcomes and use measures, categorisation was not always clear-cut. Hip joint replacement, for example, could be assessed as curative or potentially avoidable service use. Also, some variables such as waiting time for a physician’s appointment may reflect both a use measure as well as an access variable. These issues already posed a challenge during the screening process, when selecting the included studies.

We also encountered heterogeneity in the measurement of predictor variables. Our income category includes categorical and metrical income measures, individual-level variables, household-level and aggregated regional level variables.

Another limitation stems from the strong heterogeneity in statistical methods chosen by the included publications. This is the reason for choosing the harvest plot to illustrate our results over any kind of pooling.

## Conclusions

Our results confirm that socioeconomic variables and access factors play a crucial role in healthcare use. Additionally, we find evidence on interaction effects between socioeconomic and access factors on healthcare use, although research on this topic is sparse. Access variables most often investigated in the included studies comprised density measures such as physician and hospital densities. Further factors such as office-hours, working hours, and transportation to health facilities were additionally perceived as barriers to healthcare use.

### Supplementary Information

**Supplementary Material 1.**

## Data Availability

All data generated or analysed during this study are included in this published article [and its supplementary information files].
