# Supplementary material for: The role of mesolevel characteristics of the health care system and socioeconomic factors on health care use – results of a scoping review
Source: Int J Equity Health. 2024 Feb 23;23:37. doi: 10.1186/s12939-024-02122-6 (PMC10885500; doi:10.1186/s12939-024-02122-6)
Supplement: Supplementary file 1 — Supplementary Material 1. [file 12939_2024_2122_MOESM1_ESM.zip › Appendix_table1.docx]

Table A1: Overview on descriptors used in search. Descriptors were searched in the title and abstract fields. Terms that were also used as MeSH terms are denoted in italic font.

| Search block ‘Health Care Use’: | Search block ‘Access’ | Search block ‘Socioeconomic position’ |
| --- | --- | --- |
| (descriptors within search block are connected with OR):   - Health services underuse - Health care seeking behaviour - *Health services needs and demand* - *Delivery of health care* - *Medical overuse* - Health services overuse - Health services overutilization - Health care utilization - Health services utilization - *Physician* visits - *Pediatrician* visits - Children’s doctor visits - Baby doctor visits - *Referral and consultation* | **Two sub-blocks:**  Sub-block ‘regional’ AND  Sub-block ‘access measures’  Sub-block ‘regional’:  (descriptors within search block are connected with OR):   - Region - Neighbourhood - Geographic - Local - Spatial - Borough   Sub-block ‘access measures’:  (descriptors within search block are connected with OR):   - *Health services accessibility* - Access to health care - Availability of health services - *Travel time*s - *Travel* distance - Waiting *time*s - *Hospital bed*s - *Physician* density - *Pediatrician* density - *General practitioner* density - Health care supply - Office hours - Consultation hours | (descriptors within search block are connected with OR):   - Economic level - Assets index - SEP - *Health status disparities* - *Health equity* - *Social determinants of health* - *Healthcare disparities* - Health care inequalities - Socioeconomic position - *Socioeconomic factors* - *Social class* - Socioeconomic status - Social gradient - Inequity - Inequality - Gap - *Poverty* - Deprivation - *Education* - *Educational status* - *Income* - *Family income* - Schooling |
