# Supplementary material for: The role of mesolevel characteristics of the health care system and socioeconomic factors on health care use – results of a scoping review
Source: Int J Equity Health. 2024 Feb 23;23:37. doi: 10.1186/s12939-024-02122-6 (PMC10885500; doi:10.1186/s12939-024-02122-6)
Supplement: Supplementary file 1 — Supplementary Material 1. [file 12939_2024_2122_MOESM1_ESM.zip › Appendix_table2.docx]

Table A2: Full list of included studies

| **Author (Year)** | **Country** | **Data year** | **Study population** | **Sample size** | **Methods** | **Outcome: positive** | **Outcome: negative** | **Outcome: neutral** | **SEP measure** | **Access measure** | **Focus on mediation** |
| --- | --- | --- | --- | --- | --- | --- | --- | --- | --- | --- | --- |
| Field / Briggs (2001) | UK | 2000 | children, adults (diabetics, asthmatics) | 592 | χ2 tests |  |  | frequency of primary care utilisation | S | D, R |  |
| Maheswaran et al. (2003) | UK | 2000 | children, adults (15-84 years) | 998 patients on hemodialysis, 550 on peritoneal dialysis, and 1075 with renal transplants | Poisson regression |  |  | renal replacement therapy (haemodyalisis; peritoneal dialysis; transplantation) | P* | D* |  |
| Woods et al. (2003) | USA | 1999-2000 | children (<5 years; ≥5 years) | 305 households | log-linear regression |  |  | health care visits | I, E, I* | D |  |
| Arcury et al. (2005) | USA | 1999-2000 | adults (>18 years) | 1059 | parsimonious multivariate survey log-linear regression models | health care visits for regular check-up |  | health care visits for chronic care; health care visits for acute care | I, E, S, W, I*, W* | P*, D*, R* |  |
| Kirby / Kaneda (2005) | USA | 2000 | adults (≥ 18 years, women) | 25096 | logistic regression |  | having an unmet medical need |  | I*, P*, E*, W* | P*, T* |  |
| Vanasse et al. (2005) | Canada | 1999-2000 | adults (women / men, ≥ 65 years) | 25852 | logistic regression |  |  | bone mineral density testing | I, I* | D, D* |  |
| Chaix et al. (2005) | France | 1998 | adults (> 65 years) | 10995 | multilevel regression |  |  | specialists visits in relation to PCP visits | X | P, S |  |
| Kirby / Kaneda (2006) | USA | 2000 | adults (> 25 years) | 22656 | logistic regression |  | having poor access to health care |  | I*, P*, E*, W* | P*, S*, T* |  |
| Carruth et al. (2006) | USA | n.n. | adults (≥ 18 years, women) | 2324 | multiple logistic regression |  |  | failure to obtain cervical cancer screening | E, W, E*, W* | R, R* |  |
| Giorda et al. (2006) | Italy | 2001 | adults (20–75 years) | 3457 | two-level generalized hierarchical linear regression |  | ED visits; re-admissions for diabetes-related complications; hospital visits |  | E, E* | P, H* |  |
| Ionesu-Ittu et al. (2007) | Canada | 2000-2001 | adults (elderly, ≥ 65 years) | 95173 | χ2 tests, Wilcoxon rank sum test, Poisson regression |  | ED visits |  | P, P* | P, D, P*, D* |  |
| Cadarette et al. (2007) | Canada | 2003-2004 | adults (65-89 years) | 871 | logistic regression |  |  | DXA testing; treatment (alendronate, etidronate, risedronate, calcitonin, and/or raloxifene; yes or no) | I, E, I*, E* | T, T* |  |
| Harris et al. (2008) | USA | 2000 | population | 1265414 | univariable and multiple linear regression |  | myocardial infarct hospitalisations; heart failure hospitalisations |  | I, P, E, S, I*, P*, E*, S* | P, D, P*, D* |  |
| Penfold et al. (2008) | USA | 2001-2003 | children (2-20 years) | 8086 | χ2 analyses, ANOVA, hierarchical, cross-classified, generalized linear regression |  | perforated appendicitis |  | P* | D*, V*, U* |  |
| Jewett et al. (2018) | USA | 1995–2007 | adults (50-74 years, women) | 6075 | proportional odds and logistic regression | mammography screening |  |  | I, E | S, R, S*, R* | x |
| Judge et al (2009) | UK | 2002 | adults (≥ 50 years) | ~ 55 m. | multilevel Poisson regression |  |  | hip / knee replacement | E, E* | D, V, U, D*, V*, U* |  |
| Magner et al. (2009) | USA | 2003 | Medicaid enrollees | 28767985 | bivariate analysis, multiple regression |  |  | carotid endarterectomy utilisation | I*, P*, E*, S* | P*, S*, T* |  |
| Meersman et al. (2009) | USA | 2001 | adults (40–84 years, women) | 4249 | logistic generalized linear mixed effects | mammography screening |  |  | I, E, W, I*, E, W* | S* |  |
| Sørensen et al. (2009) | Denmark | 2006 | all inhabitants | ~ 5.4 m. | multiple ordinary least squares regression |  | referrals to outpatient hospital treatment; inpatient hospital treatment | referrals to private specialists | I, E, S, I*, E*, S* | S*, T* |  |
| Mobley et al. (2009) | USA | 2002–2003 | adults (65-104 years, women) | 70129 | multilevel probit regression | mammography screening |  |  | P*, W*, M* | P*, S*, D* |  |
| Chen et al. (2009) | USA | 2002 | adults | 442 rural hospitals | generalized estimation equation regression |  | hospitalisations due to ambulatory care sensitive conditions |  | I*, S* | P*, T*, V*, O* |  |
| Knudson et al. (2009) | USA | 2001-2004 | children (2-17 years) | 6 geographically diverse states | multivariate regression |  | hospitalisations for asthma |  | P | p* |  |
| Petrelli et al. (2010) | Italy | 2003 | population | ∼4,2 m. | hierarchical regression |  | hospitalisations | out-patient care utilisation; pharmaceutical care utilisation | E*, S*, W*, M* | T*, D* |  |
| Concannon et al. (2009) | USA | 2004 | adults (≥ 18 years) | 5887 | multivariate regression |  | elapsed time in emergency medical services; delay in emergency medical services |  | I*, M* | D* |  |
| Gage et al. (2009) | UK | 2003-2004 | adults (cancer patients) | 761 | stepwise logistic regression |  |  | complementary and alternative medicine utilisation | E* | D* |  |
| Rosato et al. (2009) | Italy | 2000-2004 | adults (Incident breast cancer patients) | 16022 | multinomial logistic regression |  | breast-conversing therapy surgery with/without radiotherapy; mastectomy |  | E*, W* | D*, V* |  |
| Haas et al. (2010) | USA | 2000, 2003 | adults (≥ 50 years) | 23229 | multi-level logistic regression | colorectal cancer screening |  |  | I*, E* | P*, S* |  |
| Patel et al. (2010) | USA | 2005 | adults (≥ 45 years) | 293 | binary logistic regression | prostate cancer screening |  |  | I, E, S, W | P, D, H |  |
| Rubin et al. (2011) | Denmark | 2009 | adults (40–90 years) | 4194 | logistic regressions |  |  | use of DXA scanning | I*, S*, W*, M* | D, D* |  |
| Tonner et al. (2010) | USA | 2004-2007 | adults (≥ 18 years) | 755 | multivariate regression |  |  | physician visits for systemic lupus erythematosus | E, P, E*, P* | S, S* |  |
| Zulian et al. (2011) | Italy | 2000-2006 | adults ≥ 14 years) | 12347 | Poisson regression |  | mental health services use in hospitals | mental health services use | S, W | D, D* |  |
| Diaz-Granados et al. (2010) | Canada | 2001 | children, adults (≥ 15 years) | 36984 | multilevel multivariable logistic regression |  |  | use of general practitioner–family physician services for mental health care; psychiatric services for mental health reasons | I*, E*, S*, W*, M* | P, S, P*, S* |  |
| Guttmann et al. (2010) | Canada | 2001, 2003-2005 | children (0 - 17 years) | 1042 | logistic and Poisson regression |  | hospitalisations due to ambulatory care sensitive conditions; ED visit; no preventative care visits; no primary care visits; no newborn visit | use for discretionary conditions | I* | P, P* |  |
| Bronstein et al. (2011) | USA | 2001–2006 | adults (mothers covered by Arkansas Medicaid) | 5150 | logistic regression |  |  | likelihood of infant delivery at NICU | P*, E*, W* | S*, D* |  |
| Patel et al. (2012) | USA | 2005 | adults (≥ 50 years) | 460 | binary logistic regression | colorectal cancer screening |  |  | I, E, S, W | P, D, H |  |
| Telleen et al. (2012) | USA | 2010 | children (4-8 years) | 320 | bivariate analysis, linear, Poisson, multiple logistic regression |  |  | frequency of dental visits; continuity of care; initiation of care | I, E, I* | H, H* |  |
| Smith et al. (2011) | USA | 2010 | adults (≥ 41 years, women) | 1242 | multinomial logistic regression | mammography screening |  |  | E, E* | P, P* |  |
| Margolis et al. (2011) | USA | 2006-2008 | children, adults (full population of U.S. Medicare beneficiaries with diabetes) | ~ 5 m. | geographically weighted regression |  | lower-extremity amputation |  | X, X* | P, P* |  |
| Haroon et al. (2011) | UK | 2009-2010 | children, adults (residents who received an antiviral drug for influenza-like illness) | 10655 | multivariable regression |  |  | antiviral drug collection | P, P* | D, D* |  |
| Judge et al. (2012) | UK | 2007 | adults (≥ 20 years) | 354 local authority districts | multilevel Poisson regression |  |  | renal replacement therapy | P, P* | S, R, S*, R* |  |
| Magán et al. (2011) | Spain | 2001-2003 | adults (≥65 years) | 34 health districts | Poisson regression |  | hospitalisations due to ambulatory care sensitive conditions |  | I*, E*, W* | P*, R* |  |
| Pracht et al. (2011) | USA | 1999-2006 | individuals in counties | 58 | linear regression |  | avoidable hospitalisations |  | P*, S* | P*, D*, O* |  |
| Hsia et al. (2011) | USA | 2007 | individuals treated in hospitals | ~ 9.2 m. | ANOVA, χ2, logistic regression |  | leaving the ED without being seen |  | I, I* | O*, T* |  |
| Akinyemiju et al. (2012) | USA | 2008 | adults (50-74 years, women) | 1163 | multilevel logistic regression | mammography screening; clinical breast examination |  |  | I, E, S, I*, E*, S*, M*, X* | P*, T* |  |
| Barner et al. (2010) | USA | 2002 | adults (≥ 18 years) | 2952 | multivariate logistic regression |  |  | complementary and alternative medicine utilisation | I*, E*, S*, W* | P* |  |
| Grillo et al. (2012) | France | 2005 | adults (≥ 18 years, women) | 1843 | multilevel logistic regression |  | absence of cervical cancer screening |  | I, P, E, S, M, E*, S*, M* | P, P* |  |
| McCall-Hosenfeld et al. (2012) | USA | 2004-2005 | adults (18-45 years) | 1420 | χ2 tests, random intercept partial proportional odds generalized linear mixed models | screening and vaccination index; preventive counselling index |  |  | P, E, P*, E* | P, T, P*, T* |  |
| Ryvicker et al. (2012) | USA | 2008 | adults (60-99 years) | 1260 | logistic regression |  |  | outpatient care utilisation | E* | P, P* | x |
| Borda-Olivas et al. (2013) | Spain | 2006 | adults (> 65 years) | 221214 admissions, 9923 avoidable hospitalisations | spatial regression |  | avoidable hospitalisations |  | X* | D, D* |  |
| Goswami et al. (2012) | USA | 2008-2009 | adults (>17 years) | 496 | log binomial models with backward elimination |  |  | latent tuberculosis infection treatment initiation; completion | E, P*, E*, S* | D |  |
| Harrington et al. (2012) | Canada | 2006, 2008 | adults | 1635 | logistic regression |  |  | realized access to PCPs | E*, S*, W* | P* |  |
| Sacerdote et al. (2012) | Italy | 2000-2007 | adults (incident colorectal cancer patients) | 24187 | random-intercept logistic regression |  | postoperative in-hospital mortality | proportion of preoperative radiotherapy; proportion of abdominoperineal resection | E*, W* | S*, D*, V* |  |
| Archibald / Rankin (2013) | USA | 2004 | individuals in U.S. counties | 3141 | spatial regression |  |  | substance abuse disorder assessment, assessment of other mental health problems | I*, E* | P*, T* |  |
| Blain et al. (2014) | UK | 1997-2007 pneumonia, 1995-2010 empyema | children (0–14 years) | 3874 | bayesian conditional autoregressive models |  | hospitalisations for pneumonia |  | P, P*, E* | D*, D* |  |
| Butler et al. (2013) | Australia | 2003-2009 | children (0-4 years) | 79 local government areas | bivariate analysis |  | hospitalisations due to ambulatory care sensitive conditions: total, asthma, dental, ear, nose and throat infections, gastroenteritis and dehydration, influenza and pneumonia, other vaccine preventable conditions |  | X | P |  |
| Cavalieri (2013) | Italy | 2006 | adults (≥ 18 years) | 45175 | multivariate logistic regression |  | having a self-reported unmet medical need |  | E, I, W | accessability | x |
| Cook et al. (2013) | USA | 2001-2003 | adults (≥ 18 years) | 12241 | random intercepts multi-level logistic regression |  |  | mental health service utilisation | P*, E*, S*, W* | S |  |
| Harrington et al. (2013) | Canada | 2010 | children, adults (> 12 years) | 21526 | multivariate logistic regression |  | reporting difficulty accessing specialist care |  | I, E, W, M | D | x |
| Kopetsch / Schmitz (2014) | Germany | 2008 | individuals in German counties | 413 | linear regression |  |  | ambulatory cases at GPs; specialists; physiotherapists | I*, E*, S*, W*, M* | P*, T* |  |
| Rudge et al. (2013) | UK | 2007-2008 | children (5-15 years), adults (≥ 15 years) | 1413363 | negative binomial regression |  | ED visits (children / adults) |  | P* | D* | x |
| Schäfer et al. (2013) | Germany | 2005 - 2009 | individuals in German counties | 407 | spatial error regression |  |  | hip / knee replacement | P* | S* |  |
| Tao et al. (2013) | Canada | 2004-2007 | individuals in 47 major cities/towns in Ontario | >40,000 | partial least squares-based structural equation modeling |  | cardiac surgery utilisation |  | E | D |  |
| Bocquier et al. (2013) | France | 2008-2010 | adults (18-64 years) | 316412 | multilevel logistic regression |  |  | New / long antidepressant treatment | P, P* | P, T, P*, T* |  |
| Willems et al. (2013) | Belgium | n.n. | children, adults | 7723 | χ2 tests, logistic regression |  | visit in ED rather than Primary Care Center |  | P, P* | D, D* |  |
| Lemstra et al. (2013) | Canada | 2007-2011 | adults (with ischemic heart disease) | 219.195 | odds ratios |  |  | cardiac rehabilitation attendance (exercise component; completion) | I | D | x |
| Bielefeldt (2013) | USA | 2007-2010 | individuals in U.S. states | 46 | Spearman, stepwise forward regression |  | admissions for gastroparesis | endoscopies; gastrostomies; nutritional support | I, P | P, S, V, O |  |
| Mathison et al. (2013) | USA | 2003-2006 | children (0-13 years) | 52110 | multivariate linear regression |  | non-urgent ED visits |  | I | P*, D* |  |
| Neri et al. (2013) | Italy | 2008 | adults (≥ 18 years) | 1238 | χ2 or Wilcoxon test, random intercept mixed-effect logistic regression |  |  | transplant waiting list activation | E, W | P, T, D, R, O, U |  |
| Jensen et al. (2014) | Denmark | 2008-2009 | adults (50-70 years, women) | 149234 | generalized linear regression | mammography screening |  |  | I, E, W | D, D* |  |
| Yasaitis et al. (2013) | USA | 2006-2007 | Medicare beneficiaries, cardiologists, and primary care physicians | 1229 | regression analysis |  |  | outpatient care utilisation | E | P* |  |
| Hadlock et al. (2013) | Canada | 1997-2007 | adults (≥ 18 years) | 1079259 | χ2 testing, multivariate logistic regressions |  |  | open-access colonoscopy | I, I* | V, U, V*, U* |  |
| Basu / Mobley (2014) | USA | 1995-2005 | adults (≥ 65 years) | 1010 | ordinary least squares regression |  | hospitalisations due to ambulatory care sensitive conditions |  | I*, P* | P*, T*, D* |  |
| Chamberlain et al. (2014) | USA | 1999-2010 | children (0-18 years) | 103961 pediatric oncology discharges | multivariate analyses |  |  | inpatient utilisation of pediatric cancer specialty centers | I, I* | D, D* |  |
| Charland et al. (2014) | Canada | 2009 | population in Montreal | 741237 vaccinations | multivariable regressions | Influenza A/H1N1p vaccinaction |  |  | P* | P*, R* |  |
| Huang et al. (2014) | USA | 1996-2006 | children, adults | 79231 | multivariable models |  |  | utilisation of high-volume hospitals for colorectal cancer | P*, E*, S* | D, D* |  |
| Luo et al. (2014) | USA | 2004-2005 | adults (≥18 years with diabetes) | 46806 | multilevel models | receipt of 7 preventive care services (influenza vaccination; pneumococcal vaccination; doctor's visit; A1C test; foot examination; eye examination; self-care education) |  |  | I, E, S, W, I*, E*, S*, W* | P, P* |  |
| Marino et al. 2014 | Australia | 2008 | adults (≥55 years) | 226 | ANOVAs, logistic regression | dental care utilisation |  |  | I, E, W | W |  |
| Ouedraogo et al. (2014) | France | 2010-2011 | adults (51–74 years) | 13565 | univariable logistic regression, multilevel multivariable logistic regression | mammography screening |  |  | X, X* | D, D* |  |
| Ozegowski / Sundmacher (2014) | Germany | 2010 | individuals in German districts | 412 | weighted least squares regression |  |  | equity index (degree of disparity between need for and actual utilisation of outpatient health services) | I*, P* | P*, S* |  |
| Patel et al. (2014) | USA | 2005 | adults (≥ 40 years, women) | 334 | binary logistic regression | mammography screening; clinical breast examination |  |  | I, E, S, W | P, D, H |  |
| Weeks et al. (2014) | France | 2009 - 2010 | adults (45-99 years) | 138199 admissions for knee replacement; 169156 admissions for hip replacement; 169387 admissions for hip fracture | spatial regression analysis |  | admissions for hip fracture | hip / knee replacement | I*, S* | T |  |
| White et al. (2014) | UK | 2006-2010 | individuals with mental illnesses | 162410 | negative binomial regression |  | admissions for severe mental illness |  | P* | D* |  |
| Vogt et al. (2014) | Germany | 2008-2011 | individuals in German districts | 402 | ordinary least squares and spatial lag regression | prostate cancer screening; cervical cancer screening; colon cancer screening; skin cancer screening; mammography screening |  |  | I*, E* | P*, S*, D* |  |
| Widdifield et al. (2014) | Canada | 2000-2009 | adults (newly diagnosed patients with RA) | 19760 | hierarchical logistic regression |  |  | rheumatology visits | I, I* | S, D, S*, D* |  |
| Eibich / Ziebarth (2014) | Germany | 2006, 2008, 2010 | adults (17-100 years) | 23167 | ordinary least squares regression |  | hospital use | ambulatory doctor visits | I, E, S, W, I*, E*, S*, W* | P*, T* |  |
| Gusmano et al. (2014) | France | 2004-2008 | adults (>20 years) | hospitals in 2 regions (Nord-pas-de-Calais, Provence-Alpes-Côte d' Ázur; Ile de France) | generalized linear mixed effect models |  | hospitalisations due to ambulatory care sensitive conditions | rates for revascularization – bypass surgery and angioplasty | I*, E* | P*, O* |  |
| Herrin et al. (2015) | USA | 2007-2010 | adults (discharged with myocardial infarction, heart failure, or pneumonia) | 4073 hospitals | hierarchical linear models |  | hospital readmissions |  | I, P, E, S, W, P*, E*, W* | P, S, T, P*, S*, T* |  |
| Kottwitz (2014) | Germany | 2006-2011 | children (newborns) | 707 | logistic regression |  | caesarean section |  | E, E*, M* | T, T* | x |
| Henry et al. (2014) | USA | 2008, 2010 | adults (40–74 years, women) | 5197 | logistic regression | mammography screening |  |  | I, E, W, I*, E*, W* | P, D, P*, R* |  |
| Hunold et al. (2014) | USA | 2010 | adults (≥ 65 years) | 640086 | multivariable spatial error regression |  | ED visits |  | E* | P*, D* |  |
| Alruwaily et al. (2015) | USA | 2003-2012 | adults (> 17 years) | 23537 | hierarchical generalized linear regression |  |  | follow-up testing for nephrolithiasis | P, E | P, S, P*, S* |  |
| Annequin et al. (2015) | France | 2007-2008 | adults (30-79 years) | 7290 | multilevel logistic regression |  |  | reimbursement of antidepressants, private psychiatrist visits | I, E, W, M, I* | P* |  |
| Badley et al. (2015) | Canada | 2007-2008 | adults (>18 years) | 105 health planning areas | multilevel Poisson regression |  |  | arthritis office visits | X* | P*, S* |  |
| Dumas / Polk (2015) | USA | 2012 | children (15 months-5 years) | 164 | Fisher’s exact, χ2, or t-tests | dental care utilisation |  |  | I, P, E | D |  |
| Mercier et al. (2015) | France | 2012 | population | 65129298,3 | multilevel mixed regression |  | avoidable hospitalisations |  | I*, E* | P*, S*, T* |  |
| Sakai et al. (2015) | Japan | 2010 | children (< 15 years) | 47 prefectures with 1742 municipalities (128100000) | multivariate logistic regression | diphtheria, pertussis, tetanus, measles vaccination |  |  | I* | S, P*, S* |  |
| Slaunwhite (2015) | Canada | 2002 | children, adults (≥ 15 years) | 4134 | logistic regression |  | barriers to mental health care |  | I | available barriers | x |
| Toivakka et al. (2015) | Finland | 2012 | adults (with type 2 diabetes) | 9606 | χ2 test, logistic regression | hemoglobin A1c testing |  |  | I*, E*, S* | D* |  |
| Pasnisinova et al. (2016) | Czech Republic | 2008-2013 | adults (patients underwent heart transplantation) | 496 | linear regression |  |  | heart transplantation | I*, S* | D* |  |
| Arnaout et al. (2015) | Canada | 2003-2012 | adults (> 19 years) | 53015 | multivariate logistic regression |  | mastectomy vs. no mastectomy; contralateral prophylactic mastectomy vs. no contralateral prophylactic mastectomy | preoperative breast MRI utilisation | I* | V*, T* |  |
| Alvarez et al. (2017) | USA | 2002-2011 | children (0-18 years) | 24559 | logistic regression |  |  | use of pediatric cancer specialty center | I* | D* |  |
| Chew et al. (2016) | Australia | 2011 | individuals in 61 Medicare locals | 23500000 | partial correlations |  |  | coronary angiography rate | P, S | P, D |  |
| Doumouras et al. (2016) | Canada | 2009-2012 | adults (≥ 18 years) | 4990 | ordinal logistic regression |  |  | bariatric surgeries | P* | T*, D* |  |
| Fisher-Owens et al. (2016) | USA | 2007 | children (2-17 years) | 91642 | logistic regression |  | absence of a preventive dental visit |  | I*, W* | P* |  |
| Okafor et al. (2016) | USA | 2012 | patients with inpatient colonoscopies | 217055 | logistic regression |  |  | inpatient colorectal stent utilisation | I* | V, T, V*, T* |  |
| Chou et al. (2016) | USA | 2006-2010 | adults (≥ 40 years) | 117295 | multilevel regression | dilated eye examination; eye care visits |  |  | I, E, S, I*, E*, S* | S, S* |  |
| Fishman et al. (2018) | USA | 2007-2011 | adults (18–87 years) | 560000 | multilevel logit regression |  | ED visits for preventable conditions |  | I*, E* | P*, D* |  |
| Leinonen et al. (2017) | Norway | 2008–2012 | adults (25-69 years, women) | ~ 1.3 m. | modified Poisson regression | cervical cancer screening |  |  | I, E, S, W, M, I*, E*, S*, W*, M* | D, D* |  |
| Posthumus et al. (2016) | Netherlands | 2000-2008 | adults (> 20 years, 1.532.441 singleton pregnancies) | 1.532.441 | logistic multi-level model |  | labour in non-breech term and post-term pregnancies | referral during pregnancy from community midwife to obstetrician; elective caesarean section in term and post-term breech pregnancies; birth setting in low-risk pregnancies | X* | T* |  |
| Sheringham et al. (2017) | UK | 2004-2012 | individuals in administrative areas | 316 | linear models with fixed and random effects |  | hospitalisations due to ambulatory care sensitive conditions |  | P* | P* |  |
| Kelly et al. (2017) | UK | 2007-2011 | adults (women, born in Bradford cohort) | 12286 | negative binomial regression |  |  | GP visits | P, P* | P* |  |
| Lee et al. (2016) | USA | 2008-2012 | individuals in census tracts | 4797 | multivariate analysis |  | ED visits |  | P* | P*, D* |  |
| Rowe et al. (2016) | USA | 2010 - 2012 | adults (≥ 18 years) | 342; 316 | Wilcoxon rank-sum tests, multivariable negative binomial regression |  | opioid overdose deaths | overdose reversals | I, I* | D, D* |  |
| Fusco et al. (2016) | Italy | 2014 | adults (≥ 18 years) | 19000 | logistic regression |  | avoidable hospitalisations |  | E, E* | V* |  |
| Alcala et al. (2018) | USA | 2007–2012 | children (0–14 years) | 37455 ED visit, 7329 hospital admissions | Poisson multilevel regression |  | avoidable asthma related hospitalisation; preventable asthma related ED visits |  | P* | P*, D*, T* |  |
| Chalmers (2017) | USA | 2010–2013 | population of Maryland | ~ 5.7 mio. | fixed effects logistic regression |  | ED discharges for dental conditions |  | I, I* | P* |  |
| Doumouras et al. (2017) | Canada | 2008-2015 | adults (≥ 18 years) | 20982 | multilevel ordinal logistic regression |  |  | bariatric surgeries | I* | T*, D* |  |
| Feng et al. (2017) | USA | 2010 | adults (≥ 18 years) | 30423 | ordinary least squares regression | dental care utilisation |  |  | I, I* | P, P* |  |
| Finley et al. (2017) | USA | 2012-2013 | adults (veterans) | 1128 | multinomial logistic regression |  |  | post-traumatic stress disorder care | I, E, S, W | T |  |
| Fujita et al. (2017) | Japan | 2012 | adults (40-74 years) | 166966 | three-level logistic regression | utilisation of health check-ups |  |  | I, I* | P, D, P*, D* | x |
| Héquet / Rouzier (2017) | France | 2011-2013 | children (10-19 years, females) | 121636 | multivariate logistic regression | vaccinations |  |  | I, S, M, S*, M* | P |  |
| Jabo et al. (2017) | USA | 2003-2012 | children, adults (≥15 years) | 3221 acute lymphoblastic leukemia, 10029 acute myeloid leukemia | Poisson regression |  |  | cancer-directed chemotherapy status; hematopoietic cell transplantation | P, W, P*, W* | D, D* |  |
| Rommel / Kroll (2017) | Germany | 2008–2011 | adults (18–79 years) | 8152 | multilevel logistic regression |  |  | physical therapy utilisation | E, M, I*, E*, W* | P, P* |  |
| Ruhnke et al. (2017) | USA | 2004-2009 | individuals (enrolled with a lower gastrointestinal bleeding) | 34344 | logistic regression |  |  | esophagogastroduodenoscopy utilisation | E* | S*, V* |  |
| Schmidt et al. (2018) | USA | 2009-2011 | adults (≥ 18 years) | 6,603,146 in hospitals; 21,011,958 in EDs | Pearson correlations, linear regression |  | avoidable ED visits, avoidable hospitalisations |  | I, P, E, I*, P*, E* | P, S, P*, S* |  |
| Klitkou et al. (2017) | Norway | 2008–2010 | children (1-9 years) | 9309 | Poisson regression |  | hospital admissions | outpatient visits | E*, W*, M* | R* |  |
| Lines et al. (2017) | USA | 2009-2011 | children, adults (enrollees with commercial insurance) | 64623 | multivariable logistic regression |  | ED visits; ED visits due to ambulatory care sensitive conditions |  | P, P* | D* |  |
| Noah (2017) | USA | 2005-2009 | adults (women who had a live birth in 2008) | 430968 | multilevel logistic regression |  | inadequate use of prenatal care |  | P, E, W, M, X, P*, E*, W*, M*, X* | P*, T* |  |
| Collins et al. (2018) | Australia | 2009-2014 | adults (women with breast cancer) | 1213 | Mann–Whitey U test, χ2 tests, logistic regression |  | mastectomy cases |  | X | D |  |
| Cook et al. (2017) | USA | 2004–2009 | adults (≥ 18 years) | 10399 | random intercepts multi-level linear regression |  |  | initiation of mental health care; number of days of treatment during episode among those receiving any mental health treatment | I*, E*, S*, W* | p*, S* |  |
| Maeda et al. (2018) | Japan | 2013 | adults (women) | 190361 | multiple regression models |  | caesarean section |  | I, E, I*, E* | S, T, S*, T* |  |
| Packness et al. (2017) | Denmark | 2013 | adults (20–64 years) | 50636 | logistic, Poisson regression |  | mental health care utilisation (psych. emergency clinic; admissions) | mental health care utilisation (outpatient psychiatrist; psychologist; GP) | I, E | D | x |
| Abbas et al. (2017) | Germany | 2010 | children (0–17 years) | 23795 | conditional logistic regression |  |  | non‑drug psychiatric/psychotherapeutic treatment | P | P, S |  |
| Walsh et al. (2017) | USA | 1997-2012 | children (0-17 years) | ~ 3 m. | χ2 analyses, odds ratios |  |  | lingual frenotomy use | I | O, T |  |
| Carmeiro (2018) | Portugal | 2014 | individuals with hospitalisations in NHS hospitals | 924982 | ordinary least squares regression |  | hospitalisations due to ambulatory care sensitive conditions |  | I, E, I*, E* | P*, T*, D* |  |
| Delgadillo et al. (2018) | UK | 2015 | individuals in 144 IAPT providers that covered 180 local areas | 37101007,8 | weighted least squares regression |  | cases that did not receive psychological treatment |  | P* | P*, W* |  |
| Greiner et al. (2018) | Germany | 2009 | adults (> 17 years) | 19638 | negative binomial regressions |  |  | health care visits | P* | D* |  |
| Johansson et al. (2018) | Sweden | 2001-2014 | individuals in 21 Swedish regions (county councils) | 273 region-year observations (~9 Mio.) | random effects model, estimated by generalized least squares |  |  | primary care visits | I* | P* |  |
| Lavoie et al (2018) | Canada | 1994-2010 | individuals of First Nations living both on and off reserve | 96808 hospitalisations | generalized estimating equations approach |  | hospitalisations for mental health related ambulatory care sensitive conditions |  | P | P, D |  |
| Or / Penneau (2018) | France | 2013 | adults (≥ 65 years) | 757031 | multilevel regression |  | ED visits |  | I* | P*, S*, T*, D*, V*, H* |  |
| Stracci et al. (2018) | Italy | 2001-2011 | children, adults (< 80 years) | 4735 | χ2 test, logistic regression |  | receiving radiotherapy |  | P, P* | D |  |
| Viana et al. (2018) | Portugal | 2013-2014 | adults (≥ 18 years) | 939 | unconditional logistic regression |  |  | referral and completion of cardiac rehabilitation program | I*, E* | R* |  |
| Yoon et al. (2018) | USA | 2015 | adults (≥ 18 years) | 2337 | logistic regression | dental care utilisation |  |  | P, E, W, M, P*, E*, W*, M* | P, P* |  |
| Gartner et al. (2018) | USA | 2011-2013 | adults (18-44 years) | 7810 | negative binomial regression |  | hysterectomy |  | I, E, W, I*, E*, W* | S, S* |  |
| Régis et al. (2018) | France | 2012-2015 | adults (≥ 17 years) | 19466 | three level logistic regression |  |  | breast reconstruction | P, P* | S, V, O, S*, V*, O* |  |
| Shoff et al. (2019) | USA | 2012-2014 | individuals in U.S. counties | 3031 | ordinary least squares regression |  | ED admissions |  | P*, S*, M*, X* | P*, T* |  |
| Daly et al. (2018) | USA | 2013-2015 | adults (≥ 65 years) | 843 ZIP codes | ordinary least squares multivariate regression |  | avoidable hospitalisations |  | S, E | P |  |
| Patel et al. (2020) | USA | 2005 | adults (≥ 40 years, women) | 308 | binary logistic regression | cervical cancer screening, mammography screening, colorectal cancer screening |  |  | I, E, S, W | P, D, H |  |
| Ranade et al. (2019) | USA | 2013 | adults (> 18 years) | 20330 | multilevel logistic regression |  | ED visits for non-traumatic dental conditions |  | I*, P* | P* |  |
| Roy et al. (2019) | USA | 2010 | children, adults | 6 states/ 1487 ZIP codes | multivariable linear regression |  | hospitalisations |  | I* | P*, T* |  |
| Sineshaw et al. (2020) | USA | 2007 - 2014 | adults (≥ 35 years) | 179189 | generalized estimating equation models with modified Poisson regression |  |  | curative intent surgeries for early-stage non-small cell lung cancer | P, P* | S, S* |  |
| van der Goes et al. (2019) | USA | 2007-2010 | adults (18 - 100 years) | 944571 person-years | multinomial logistic regression |  |  | specialist physician visits (dementia / epilepsy / MS / parkinsonism) | I*, E*, W* | S* |  |
| Wright et al. (2019) | UK | 2011 | adults (≥ 60 years) | 294 870 | multiple log-binomial regression | funded eye examinations |  |  | P, E, W, I*, E*, W* | S, R, S* |  |
| Jayasekera et al. (2019) | USA | 2000-2007 | adults (≥ 70 years) | 37760 | χ2 test, cluster-adjusted logistic regression |  | advanced prostate cancer diagnosis |  | W, X, X* | P, P* |  |
| Shah et al. (2019) | USA | 2000-2012 | children (< 21 years) | 811941 | multivariable models |  |  | discharge with rehabilitative services | I* | V*, T* |  |
| Coyle et al. (2019) | UK | 2016 | adults (≥ 18 years) | 1272419 | logistic regression |  | uncontrolled hypertension |  | P* | V* |  |
| Okuyama et al. (2019) | Japan | 2015 | children, adults (>15 years) | 52029 | multivariable logistic regression |  | uncontrolled hypertension |  | I, I* | P, S, P*, S* |  |
| Renner (2020) | Austria | 2008 - 2013 | individuals in Austrian districts | 117 | linear fixed effects models |  | hospitalisations for acute; hospitalisations due to ambulatory care sensitive conditions |  | E*, S* | P*, S* |  |

SEP: E: education, I: income, S: employment status, M: migration status, P: poverty, W: marriage and family structure, x: composite index; Access: P: primary care density, S: secondary care density, T: tertiary care density, D: distance to provider, R: driving time to providers, W: waiting time, V: volume of health facility, O: ownership of facility, T: teaching status, H: office hours; * adjusted analysis. Studies that included more than one outcome category were noted as mixed studies in table 2 in the main text.
